# Diels–Alder Cycloaddition of Cyclopentadiene to C_60_ and Si_60_ and Their Endohedral Li^+^ Counterparts

**DOI:** 10.1021/acs.jpca.4c08287

**Published:** 2025-01-27

**Authors:** Omkar Charapale, Jordi Poater, Sergio Posada-Pérez, Miquel Solà, Albert Poater

**Affiliations:** † Institut de Química Computacional i Catàlisi and Departament de Química, 117394 Universitat de Girona, c/Maria Aurèlia Capmany 69, 17003 Girona, Catalonia, Spain; ‡ Departament de Química Inorgànica i Orgànica & IQTCUB, Universitat de Barcelona, 08028 Barcelona, Spain; § ICREA, 08010 Barcelona, Spain

## Abstract

Both silicon and carbon are elements located in group 14 on the periodic table. Despite some similarities between these two elements, differences in reactivity are important, and whereas carbon is a central element in all known forms of life, silicon is barely found in biological systems. Here, we investigate the Diels–Alder cycloaddition reaction of cyclopentadiene (CP) and cyclopentasildiene (CP_Si_) with fullerenes C_60_, Li^+^@C_60_, Si_60_, and Li^+^@Si_60_ using density functional theory methods. The results reveal distinct kinetic and thermodynamic trends that govern the reactivity and selectivity. For C_60_, the [6,6] pathway is kinetically and thermodynamically favored, whereas for Si_60_, the [5,6] pathway is preferred thermodynamically but not kinetically. The introduction of lithium cations increases the reactivity of both C_60_ and Si_60_. Energy decomposition analysis (EDA) unveils the importance of the components of the interaction energy between CP_Si_ and the corresponding fullerenes. The findings provide insights into the interplay of electronic structure, substrate reactivity, and fullerene electrophilicity in cycloaddition reactions.

## Graphical abstract

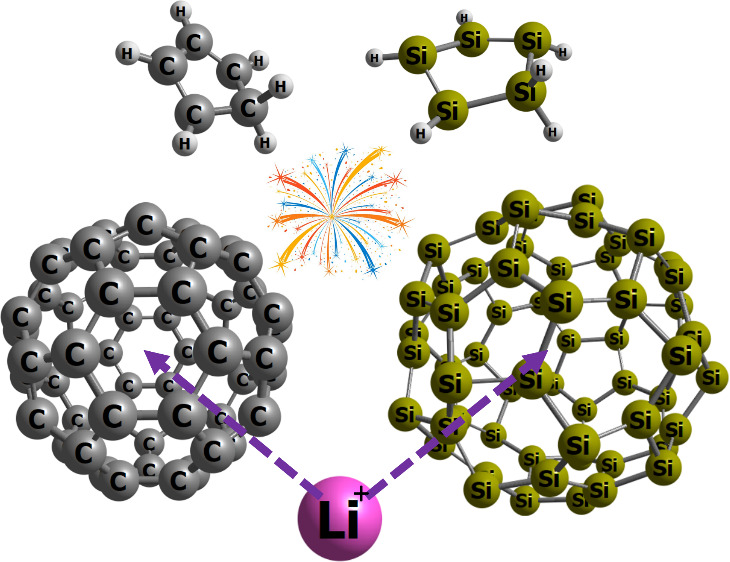

## Introduction

Fullerenes, characterized by their unique cage-like structures and exceptional chemical properties,^1,2^ have been extensively studied for their applications in nanotechnology,^3^ catalysis,^4,5^ and materials science.^6^ Among fullerenes, C_60_ represents the archetypal member of this family and has been the subject of intense research due to its high symmetry, stability, and versatile reactivity.^7^ C_60_, often referred to as buckminsterfullerene, exhibits remarkable stability derived from its delocalized π-electron system, which facilitates electron transfer processes and reactivity in cycloaddition reactions,^8,9^ such as Diels–Alder (DA) reactions.^10^ The symmetric distribution of the carbon atoms contributes to the high electron affinity and relatively low polarizability. These features make C_60_ a preferred candidate for applications in photovoltaics,^11,12^ organic semiconductors, and biomedical research.^13,14^

To expand their applicability in various fields, fullerenes must undergo functionalization to tailor their chemical properties.^15^ For example, the incorporation of C_60_ into molecular heterojunction dye-sensitized solar cells requires the attachment of a donor group to form donor–acceptor (D–A) dyads,^16,17^ which are essential for efficient charge separation and energy transfer.^18^ This has been achieved using several functionalization strategies, including cycloaddition reactions such as [4+2] Diels–Alder (DA) reactions,^19,20^ [3+2] Prato reactions,^21,22^ Bingel cyclopropanations,^23^ and [2+2+2] cycloadditions.^24^ These approaches offer highly efficient and versatile methods for modifying fullerenes, often with regioselectivity^25^ and, in some cases, enantioselectivity,^26^ enabling precise control over the resulting fullerene derivatives.

Structurally, C_60_ fullerenes feature two distinct types of bonds: pyracylenic [6,6] bonds, which connect two fused six-membered rings (6-MRs), and corannulenic [5,6] bonds, located at the junctions of five- and six-membered rings. In empty fullerenes, cycloaddition reactions predominantly occur at the more reactive [6,6] bonds,^9,27^ as these bonds exhibit higher electron density and lower strain compared to [5,6] bonds. However, in the case of endohedral fullerenes, where a foreign atom or cluster is encapsulated within the fullerene cage, the preference between [6,6] and [5,6] bonds becomes less predictable.^28,29^ The altered electronic environment and steric effects introduced by the encapsulated species can significantly influence the regioselectivity of the cycloaddition reactions, leading to varied preferences, as observed in multiple studies. Understanding and controlling this selectivity are critical for the rational design of fullerene-based materials with targeted properties for applications in energy, materials science, and medicine.

Fullerene cages can undergo multiple additions depending on the reaction conditions,^30–32^ allowing for the formation of higher-order adducts with high regioselectivity control. For instance, multiple DA cycloadditions to C_60_ can yield bisadducts, trisadducts, and further products, culminating in the formation of a T_h_-symmetric hexakisadduct with pseudooctahedral geometry through six consecutive additions.^33,34^ These DA reactions exclusively target the [6,6] bonds of C_60_. However, some of the resulting cycloadducts are thermally unstable and may undergo cycloreversion under certain conditions.^35^

In a computational study, Solà et al. investigated the formation of the T_h_-symmetric hexakisadduct via successive DA cycloadditions of 1,3-butadiene to the [6,6] bonds of C_60_.^36^ Their findings showed that with each successive addition, the enthalpy barriers slightly increase, while the exothermicity of the reactions gradually diminishes. Experimentally, it was found that the incarcerated lithium cation in [Li^+^@C_60_]­(PF_6_^–^) has a strong impact in the DA, due to the lowering of the LUMO level of the fullerene double bond by interacting with the Li^+^.^37,38^ This was confirmed computationally with the study by Das and co-workers of the successive DA cycloadditions of 1,3-butadiene to C_60_ and Li^+^@C_60_.^39^ These studies highlight how the energetic landscape evolves during these stepwise addition processes, providing valuable insights into the chemical reactivity and limitations of fullerene functionalization.

On the other hand, silafullerene Si_60_, though structurally analogous to C_60_, demonstrates notable deviations in electronic structure and bonding due to the larger atomic radius and lower electronegativity of silicon.^40^ First, the Ih geometry of C_60_ is partially lost in Si_60_. Second, the absence of delocalized π-electrons in Si_60_ results in a more localized electronic structure, leading to increased polarizability and a pronounced tendency toward stronger electrostatic interactions.^41^ Because of the instability of Si_60_, studies on Si_60_ are not based on experiments but on ab initio calculations.^42,43^ The results reveal that in these structures, some silicon atoms protrude outward, while others shift inward, leading to the formation of sp^3^ hybridization in the silicon framework,^44^ and to stabilize them, the insertion of ions and molecules leading to endohedral silafullerenes,^45,46^ metal-doped,^47^ exohydrogenated,^48–51^ or the passivation by F and Cl could be viable alternatives.^52^ Additionally, the introduction of lithium (Li^+^@Si_60_) or other substituents further modifies its electronic properties, enhancing its potential for tailored applications in catalysis and materials science.^53^ In addition, endohedral metallofullerenes can lead to magnetic properties.^54–56^

Despite the important differences between carbon- and silicon-based chemistries, there are also some similarities. Compounds such as cyclopentasilane,^57^ octasilacubane,^58^ hexasilaprismane,^59^ or cyclotrisilene^60^ among others have been synthesized and have allowed the comparison between the two chemistries. We consider that a comparative study between C_60_ and Si_60_ may provide valuable insights into how structural and electronic differences influence DA reactivity.^61^ For example, while C_60_ typically favors [6,6]-cycloaddition due to the distribution of π-electrons,^62^ preliminary investigations suggest that Si_60_ may exhibit a preference for [5,6]-cycloaddition under certain conditions. These variations are attributed to the dominance of the Pauli repulsion and orbital interaction components in Si_60_, contrasting with the balanced contributions observed in C_60_. Such differences not only expand the fundamental understanding of fullerene chemistry but also highlight the potential of Si_60_ in applications where C_60_’s properties are less advantageous.^63^

This density functional theory (DFT) study aims to delve into the comparative behavior of C_60_ and Si_60_ in representative reactions, analyzing their interaction energies, electronic contributions, and effects of the endohedral fullerene with lithium as a central cation. By elucidating these differences, the research contributes to a broader understanding of how structural and electronic factors govern the reactivity of fullerenes and their derivatives, paving the way for their strategic application in advanced materials and catalytic systems.

## Computational Details

Theoretical calculations were conducted using the Gaussian16 software package.^64^ Geometry optimizations and frequency analyses were performed with the B3LYP hybrid functional,^65,66^ employing the 6-31G­(d) basis set,^67^ and Grimme’s D3 dispersion correction with Becke–Johnson (BJ) damping.^68^ Dispersion corrections are essential to accurately model the chemical reactivity of fullerenes.^69^ For single-point energy refinements, the B3LYP functional was paired with the 6-311G­(d,p) basis set.^70^ All calculations utilized an ultrafine integration grid. Solvent effects were omitted because of the difficulty of implicit solvent models to work with encapsulated entities, apart from the low polarity of the solvents used in DA reactions,^71^ particularly toluene or dichloromethane. Thermal and entropy corrections, calculated at the B3LYP-D3­(BJ)/6-31G* level under gas phase conditions (298.15 K, 1 atm), were added to the B3LYP-D3­(BJ)/6-311G­(d,p) electronic energies to obtain the reported enthalpies and Gibbs energies.

The interaction between the fullerene and the cyclopentadiene was analyzed within the framework of Kohn–Sham molecular orbital theory in combination with a quantitative energy decomposition analysis (EDA) in the gas phase.^72–75^ EDA has been performed with the Amsterdam Density Functional (ADF) program using dispersion-corrected density functional theory at the ZORA-BLYP-D3­(BJ)/TZP//B3LYP-D3­(BJ)/6-31G* level of theory· ^68,76,77^ In the EDA, the bond energy Δ*E* between fragments is decomposed into the deformation energy, Δ*E*_def_, associated with the deformation of the two fragments from their equilibrium structure to the geometry they adopt at a given point in the reaction coordinate plus the interaction energy, Δ*E*_int_, between these deformed fragments. The interaction energy Δ*E*_int_ between these fragments is decomposed into classical electrostatic attraction Δ*V*_elstat_, Pauli repulsion Δ*E*_Pauli_ between occupied orbitals, stabilizing orbital interactions Δ*E*_oi_, and dispersion Δ*E*_disp_.

## Results and Discussion

The cycloaddition step with cyclopentadiene (CP) has been studied on fullerene C_60_ and endohedral fullerene Li^+^@C_60_ in Table 1. For C_60_, the DA of CP to C_60_ has a kinetic cost in Gibbs energies of 22.8 kcal/mol, leading to the endergonic product (1.4 kcal/mol).^78,79^ Our calculated enthalpies overestimate the available experimental data for the barrier and underestimate the reaction enthalpy.^55^ The experimental enthalpy barrier of 6.9 kcal/mol has to be compared to our calculated enthalpy difference of 13.7 kcal/mol between the transition state (TS) and the reactant complex (RC), whereas the reaction enthalpy of −19.8 ± 2.2 kcal/mol is closer to our calculated value of −14.1 kcal/mol. The large differences between enthalpy values and Gibbs energies are likely due to an overestimation of the role of entropy in the Gibbs energy.^80–82^ To note that the higher pressure suggested by Yang et al. could be a possiblet solution to decrease the activation energy and increase the exothermicity,^83^ making the DA reaction more favorable.

**Table 1:** Relative Gibbs and Enthalpy Energy Values (in kcal/mol) of a First Cycloaddition of C_60_ and Li^+^@C_60_ with Cyclopentadiene

structure	C_60_		Li^+^@C_60_	
	Δ*H*	Δ*G*	Δ*H*	Δ*G*
C_60_+cyclopentadiene	0.0	0.0	0.0	0.0
RC^a^	–4.9	4.2	–8.3	1.7
TS[6,6]^b^	8.8	22.8	0.7	14.5
product [6,6]	–14.1	1.4	–20.2	–4.7
TS [5,6]	22.0	36.4	12.4	27.1
product [5,6]	5.2	20.6	–0.9	14.4
TS [5,6]’	20.4	34.6	11.0	25.6
product [5,6]’	5.7	21.0	–0.6	14.7

a RC = reactant complex.

b TS = transition state.

The kinetics clearly favor the [6,6] attack over either of the two possible [5,6] attacks by about 10 kcal/mol (see Figure 1). The difference between the [5,6] and [5,6]' attackslies in whether the methylene group of CP is positioned over the six-membered or five-membered ring, respectively. This disadvantage is even more pronounced thermodynamically, with nearly 20 kcal/mol for both possible isomers. It is worth mentioning that the only reactant complex (RC) located is 4.2 kcal/mol above the separated fragments according to the Gibbs energy, whereas below by 4.9 kcal/mol from the enthalpy. On the other hand, with the insertion of a lithium ion into C_60_, the scenario changes: an RC is observed with an enthalpic stabilization of 3.4 kcal/mol. This increased reactivity of the fullerene continues with an energy barrier of only 9.0 kcal/mol with respect to both separated reactants and still a negligible endothermic value of 0.7 kcal/mol, in agreement with past results.^84,85^ Regarding selectivity, the best [5,6] attack remains kinetically disfavored by 10.3 kcal/mol.^39^

**Figure 1:**
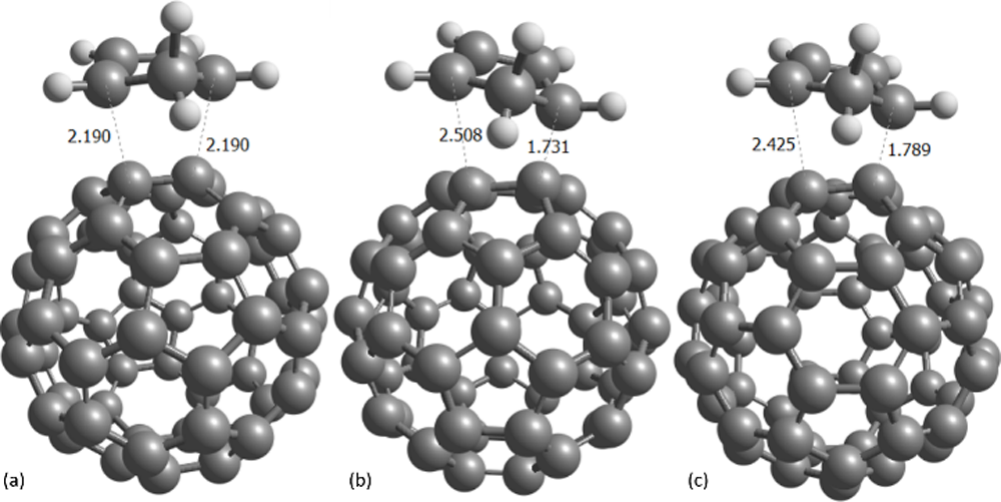
Diels–Alder transition states for C_60_: (a) [6,6] attack, (b) [5,6] attack placing the methylidene group over the five-membered ring, and (c) over the six-membered ring (selected distances in Å). The latter is the so-called [5,6]’ attack in Tables 1, 2, and 4.

Some clarifications must be made. First, the insertion of Li^+^ in C_60_ to form Li^+^@C_60_ stabilizes the LUMO, increasing significantly the reactivity of Li^+^@C_60_.^55^ In other words, the insertion of a lithium cation makes C_60_ more electrophilic. Second, the comparison of the [6,6] attack versus the [5,6] attack is hindered by the fact that the TSs are not perfectly symmetric, i.e., one C···C distance is significantly longer than the other one.

Next, C_60_ was replaced with Si_60_ to investigate the DA reaction with CP. For comparison, the carbon atoms in the substrate were also substituted with silicon. The first notable observation was that no RC could be found for Si_60_. Interestingly, the TS for the [6,6] attack of CP on Si_60_ could not be identified; instead, a TS corresponding to the rotation movement of the CP around the [6,6] bond was found, with a Gibbs energy barrier of only 5.7 kcal/mol relative to the separate reactants. The DA cycloaddition of CP to the [6,6] bond of Si_60_ was found to be barrierless. This finding confirms the high reactivity of Si_60_ as well as the free rotation of the CP when approaching silafullerene Si_60_. For the [5,6] attack, we were able to find the TSs of the DA reaction with energy barriers of 7.8 and 6.5 kcal/mol for the two isomers ([5,6] and [5,6]’, see Figure 1). Overall, these results indicate that the reactivity of fullerene is enhanced for Si_60_. However, since the [6,6] attack was found to be barrierless, we cannot conclude that the [5,6] attack is more favorable for Si_60_ compared to C_60_. The insertion of a lithium cation into Si_60_ corroborated these findings. In this case, the [5,6] attack was nearly barrierless, with TSs associated with Gibbs energy barriers up to only 0.7 kcal/mol.

Since the TSs for the [6,6] reaction could not be located for Si_60_ and no linear transit approach yielded consistent results, only the Diels–Alder products were analyzed to gain a global perspective, as collected in Table 2. Actually, the product of the reaction with Si_60_ resulting from the [6,6] attack is highly exergonic, by 21.7 kcal/mol and even more so in the presence of lithium (27.2 kcal/mol). With the substrate cyclopentadiene containing silicon atoms (CP_Si_), the exergonic effect is significantly amplified, reaching values of 28.2 and 64.5 kcal/mol for C_60_ and Si_60_, respectively. Furthermore, introducing lithium increases these values to 35.9 and 67.6 kcal/mol. Overall, upon comparison of the [5,6] and [6,6] attacks, Table 2 consistently shows that the latter is the favored DA pathway for C_60_. However, this preference is reversed for Si_60_, for which [5,6] becomes more stabilized. However, it is important to highlight that this CP_Si_ establishes a significantly stronger interaction with a novel product typology, linking both silylene (Si–H) groups of the substrate to Si_60_. This results in an overstabilization of 2.2 kcal/mol for the [6,6] product but a remarkable 23.9 kcal/mol for the [5,6] product with respect to the absence of such an interaction. Consequently, the final preference shifts to the [5,6] pathway with a difference of 21.7 kcal/mol in its favor.

**Table 2:** Relative Gibbs and Enthalpy Energy Values (in kcal/mol) of a First Cycloaddition of C_60_, Li^+^@C_60_, Si_60_, and Li^+^@Si_60_ with CP and CP_Si_

	Δ*H*	Δ*G*	Δ*H*	Δ*G*	Δ*H*	Δ*G*	Δ*H*	Δ*G*
	CP		CP_Si_		CP		CP_Si_	
	C_60_				Li^+^@C_60_			
product [6,6]	–14.1	1.4	–43.5	–28.2	–20.2	–4.7	–51.1	–35.9
product [5,6]	5.2	20.6	–26.0	–10.9	–0.9	14.4	–33.3	–18.3
product [5,6]’	5.7	21.0	–26.0	–10.9	–0.6	14.7	–33.7	–18.6
	Si_60_				Li^+^@Si_60_			
product [6,6]	–37.2	–21.7	–81.0	–64.5	–42.7	–27.2	–82.6	–67.6
product [5,6]	–32.4	–16.9	–94.9	–78.0	–37.4	–22.0	–106.5	–89.4
product [5,6]’	–32.0	–16.4	–94.5	–77.5	–36.9	–21.6	–106.7	–88.2

A brief mention of conceptual DFT follows,^86^ although to compare the disparity in reactivity between C_60_ and Si_60_, this is arguably unnecessary; simply analyzing the energies of the HOMO (H) and LUMO (L) suffices (see Table 3). For C_60_, the HOMO–LUMO gap is 2.760 eV, while for Si_60_, it is halved to 1.397 eV,^87^ which is a significant difference that supports the enhanced reactivity of Si_60_. It is worth mentioning that density functional tight-binding molecular dynamics by Schleyer and co-workers calculated the spontaneous disintegration of Si_60_ at temperatures up to 700 K.^88^ Examining the two orbitals, both exhibit nearly synchronous changes, with a destabilization of the HOMO and stabilization of the LUMO by approximately 0.6 eV in both cases, both favoring the reactivity of Si_60_ compared to C_60_.

**Table 3:** HOMO (H), LUMO (L), H-L Gap, and Electrophilicity (ω = μ^2^/2η) in eV

	without the insertion of Li^+^:				with the insertion of Li^+^:			
	H	L	H-L gap	ω	H	L	H-L gap	ω
C_60_	–5.986	–3.226	2.760	7.689	–9.329	–6.549	2.780	22.669
Si_60_	–5.393	–3.996	1.397	15.771	–7.406	–6.068	1.338	33.929
CP	–5.757	–1.706	4.051	3.438				
CP_Si_	–5.503	–3.662	1.842	11.402				

Contrary to what might be expected, the insertion of a lithium ion does not induce any significant change in the gaps, resulting in only a slight positive variation of 0.020 eV for Li^+^@C_60_ and a small decrease of 0.059 eV for Li^+^@Si_60_. Turning to Parr’s electrophilicity index,^89^ it is noteworthy that C_60_ is significantly less electrophilic, with a value of 7.689 eV compared to 15.771 eV for Si_60_. This observation explains the strong stabilization of the cycloaddition product with CP and Si_60_, relative to the destabilization with C_60_. Thus, it is necessary to remark on the nucleophilic attack of CP on the fullerene. With lithium, the same effect is observed but amplified. Interestingly, the presence of lithium significantly enhances the electrophilic character of the fullerene. This finding reinforces the high intrinsic reactivity of Si_60_, while for C_60_, the lithium ion more notably impacts its electronic properties. The charges from the Natural Population Analysis lead to values for lithium of 0.825 e for C_60_ and 0.918 e for Si_60_, respectively.

The two substrates, CP and CP_Si_, exemplify their inherent reactivity with fullerenes. Specifically, CP_Si_ exhibits a significantly lower HOMO–LUMO gap, driven by the stabilization of the LUMO.

Next, an energy decomposition analysis (EDA) was performed to understand how the CP interacts with the fullerenes, with the aim of justifying the above-discussed different reactivity between C_60_ and Si_60_. For such, two fragments have been considered: the (sila)­fullerene (with and without Li^+^) and CP or CP_Si_. For a better representation of the involved interactions, for the RC, both the (sila)­fullerene and the CP_Si_ fragments were considered in their singlet state. At difference, for both the TS and the product, the EDA was performed with the same fragments but in the triplet state because they involve the breaking of at least two bonds (Table 4). Nevertheless, both singlet and triplet calculations were carried out for all TS and products, keeping the same trend. Deformation energies, also enclosed in Table 4, support the chosen breaking of the fragments and appear not to be determinant in the trends, for which interaction energies and their components are responsible.

**Table 4:** Energy Decomposition Analysis for the Cycloaddition of CP and CP_Si_ to C_60_ and Si_60_ (in kcal/mol) and Their Corresponding Endohedral Fullerenes with a Lithium Ion^a^ ^,^ ^b^

	without the insertion of Li^+^						with the insertion of Li^+^					
	Δ*E*_int_	Δ*E*_Pauli_	Δ*V*_elstat_	Δ*E*_oi_	Δ*E*_disp_	Δ*E*_def_	Δ*E*_int_	Δ*E*_Pauli_	Δ*V*_elstat_	Δ*E*_oi_	Δ*E*_disp_	Δ*E*_def_
with CP												
C_60_RC	–6.5	12.5	–5.2 (28)	–4.0 (21)	–9.8 (51)	2.3	–10.9	17.6	–8.3 (29)	–9.0 (31)	–11.2 (40)	2.7
C_60_TS [6,6]	–104.9	145.6	–67.7 (27)	–166.4 (66)	–16.4 (7)	112.3	–108.1	131.0	–64.5 (27)	–158.3 (66)	–16.3 (7)	108.0
C_60_Product [6,6]	–153.5	535.4	–276.7 (40)	–395.4 (57)	–16.8 (3)	144.3	–159.6	516.1	–265.3 (39)	–393.7 (58)	–16.7 (3)	144.5
C_60_TS [5,6]	–98.2	236.8	–118.7 (35)	–200.6 (60)	–15.8 (5)	117.7	–110.3	255.5	–129.4 (35)	–220.3 (60)	–16.0 (5)	121.3
C_60_Product [5,6]	–145.0	562.8	–275.4 (39)	–415.8 (59)	–16.6 (2)	153.1	–151.0	540.8	–262.7 (38)	–412.7 (60)	–16.4 (2)	153.2
C_60_TS [5,6]’	–97.0	227.4	–110.7 (34)	–197.7 (61)	–16.0 (5)	115.1	–109.5	248.0	–124.4 (35)	–217.0 (61)	–16.2 (4)	119.1
C_60_Product [5,6]’	–143.3	552.3	–270.3 (39)	–408.8 (59)	–16.5 (2)	151.8	–149.4	530.6	–257.8 (38)	–405.8 (60)	–16.4 (2)	151.8
Si_60_Product [6,6]	–127.2	373.6	–218.9 (44)	–268.1 (54)	–13.9 (2)	98.6	–129.0	363.5	–213.6 (43)	–265.0 (54)	–13.9 (3)	99.7
Si_60_Product [5,6]	–129.9	374.7	–223.2 (44)	–267.0 (53)	–14.5 (3)	104.7	–131.4	368.1	–219.1 (44)	–265.9 (53)	–14.4 (3)	105.6
with CP_Si_												
C_60_Product [6,6]	–132.4	410.2	–225.9 (42)	–294.9 (54)	–21.8 (4)	86.2	–140.4	383.1	–205.2 (39)	–296.5 (57)	–21.8 (4)	86.5
C_60_Product [5,6]	–124.3	435.2	–228.6 (41)	–309.0 (55)	–21.9 (5)	93.9	–132.1	405.0	–206.4 (38)	–308.9 (58)	–21.8 (4)	94.2
Si_60_Product [6,6]	–128.9	399.1	–219.4 (42)	–286.0 (54)	–22.6 (4)	46.5	–119.2	271.2	–163.3 (42)	–204.7 (52)	–22.4 (4)	41.1
Si_60_Product [5,6]	–168.4	630.7	–320.3 (40)	–458.9 (57)	–19.9 (3)	78.3	–182.4	612.7	–312.6 (39)	–463.6 (58)	–18.9 (3)	83.3

a Computed at ZORA-BLYP-D3­(BJ)/TZP//B3LYP-D3­(BJ)/6-31G*. Δ*E*_int_ = Δ*E*_Pauli_ + Δ*V*_elstat_ + Δ*E*_oi_ + Δ*E*_disp_. In parentheses, the percentage of each term to the total attractive interactions (Δ*V*_elstat_ + Δ*E*_oi_ + Δ*E*_disp_).

b Analyses are performed taking into account the fragments with singlet multiplicity for RC and triplet for TS and product. Deformation energies have also been enclosed.

Starting with the RC for C_60_, the interaction is favorable (Δ*E*_int_ = −6.5 kcal/mol) mainly because of dispersion interactions (−9.8 kcal/mol (51%)) together with similar electrostatic (−5.2 kcal/mol (28%)) and orbital (−4.0 kcal/mol (21%)) contributions. This is consistent with C···C distances of 3.189 and 3.191 Å between C_60_ and the CP. Qualitatively, the endohedral Li^+^@C_60_ system shows no significant differences, with the CP reducing the distance to C_60_ to 3.034 and 3.035 Å. The increased Pauli repulsion (+5.1 kcal/mol) is compensated by improved electrostatic and orbital interactions, resulting in a −4.4 kcal/mol increase in interaction overall (Δ*E*_int_ = −10.9 kcal/mol). The more favorable Δ*V*_elstat_ is due to the more attractive interaction between the more positively charged Li^+^@C_60_ with CP, as supported by the molecular electrostatic potential isosurface (Figure 2). In addition, the more favorable Δ*E*_oi_ is caused by the larger charge transfer between the HOMO of the CP and the LUMO of Li^+^@C_60_ than that of C_60_ (0.11 vs 0.03 au, respectively, Figure 3). The larger < C_60_^LUMO^ | CP^HOMO^> overlap between these orbitals when Li^+^ is included also agrees with the stronger interaction (0.048 vs 0.042, with and without Li^+^, respectively).

**Figure 2:**
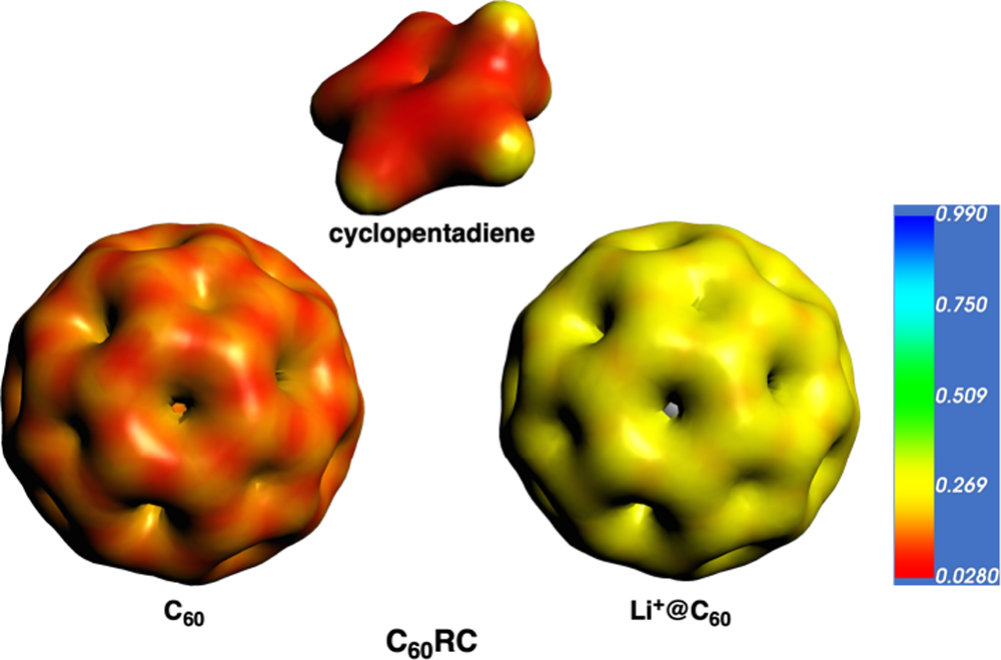
Molecular electrostatic potential isosurfaces (electronic density isovalue = 0.03 a.u.) of the fragments considered in the EDA analysis of C_60_RC, with and without Li^+^.

**Figure 3:**
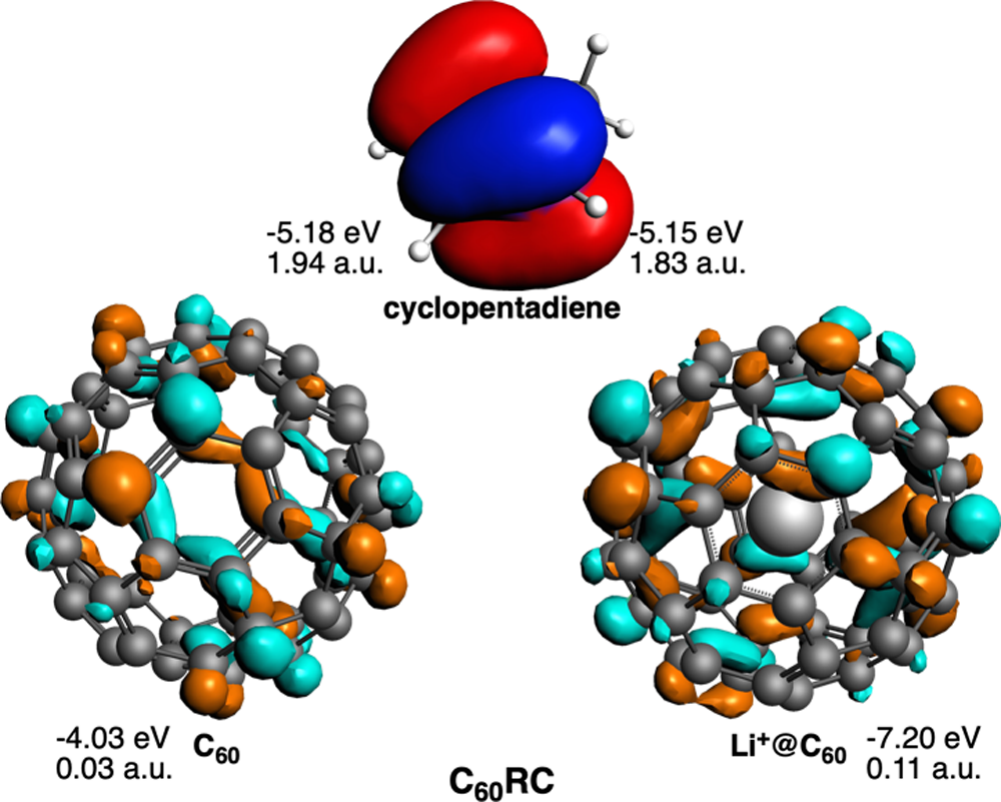
HOMO of cyclopentadiene and LUMO of C_60_ and Li^+^@C_60_ of the fragments considered in the EDA analysis of C_60_RC, with and without Li^+^. Energies of the orbitals and their gross Mulliken populations are also included.

Extending the analysis to the [6,6] TS, the differences between C_60_ and Li^+^@C_60_ become smaller. Geometrically, this is reflected in C···C distances of 2.190 Å for C_60_ and a slight increase to 2.195 Å for Li^+^@C_60_, with a reduced Pauli repulsion of 14.6 kcal/mol, leading to an overall interaction energy improvement of 3.2 kcal/mol. As expected from the Hammond postulate, a more exothermic attack goes with an earlier TS. In this case, Li^+^@C_60_ shows a stronger interaction despite both Δ*V*_elstat_ and Δ*E*_oi_ being less attractive than without Li^+^. As for RC, the charge transfer from CP to C_60_ is larger with Li^+^ (0.34 vs 0.29 au); however, Δ*V*_elstat_ does not become more attractive because of the longer distance between both fragments.

Next, with respect to the TSs, the interaction in [6,6] is stronger than either [5,6] or [5,6]’ by 6.7 and 7.9 kcal/mol, respectively, in agreement with the above-discussed data. For the [5,6] attacks, it is worth noting the highly asynchronous nature of the corresponding transition states, with C···C distances of 2.508/2.426 and 1.731/1.789 Å for C_60_. Such a shorter C···C bond length causes a large increase of the Pauli repulsion (from 145.6 to 236.8 kcal/mol), and despite such increase being partially compensated by the attractive electrostatic and orbital interaction terms compared to [6,6], this latter remains more attractive. On the other hand, with lithium cation, the same trends are also observed when going from [6,6] to [5,6], although this latter gives a slightly stronger interaction by 2.2 kcal/mol (1.4 kcal/mol for [5,6]’). This is consistent with a later TS [5,6], with C···C distances of 2.436 and 1.679 Å for the [5,6] attack.

The above EDA analysis of C_60_ supports the preferred [6,6] attack compared to the other two possible [5,6] pathways. As found for TS [6,6] and TS [5,6], product [6,6] has a stronger interaction energy by 8.5 and 10.2 kcal/mol with respect to [5,6] products. This is further evident from the sharp increase in Pauli repulsion energy for C_60_, rising from 535.4 kcal/mol (product of the [6,6]-attack) to 562.8 kcal/mol (product of the [5,6]-attack), while the electrostatic interactions remain nearly constant, with just a drop of 1.3 kcal/mol, and the orbital interactions improve from 395.4 to 415.8 kcal/mol. For the lithium system, the same rationale applies, with product [6,6] giving an 8.6 kcal/mol stronger interaction energy than [5,6] one, mainly due to an increase of 24.7 kcal/mol of Pauli for this latter, and not compensated by its more favorable orbital interaction by 19.0 kcal/mol. Thus, for both TS and product, Δ*E*_Pauli_ is clearly the determinant contribution to the interaction energy, which is directly related to the distance between both fragments. Such distance also directly influences both Δ*V*_elstat_ and Δ*E*_oi_ terms, but to a lower extent (singly occupied molecular orbitals—SOMO—for each fragment in their triplet state enclosed in Figure 4).

**Figure 4:**
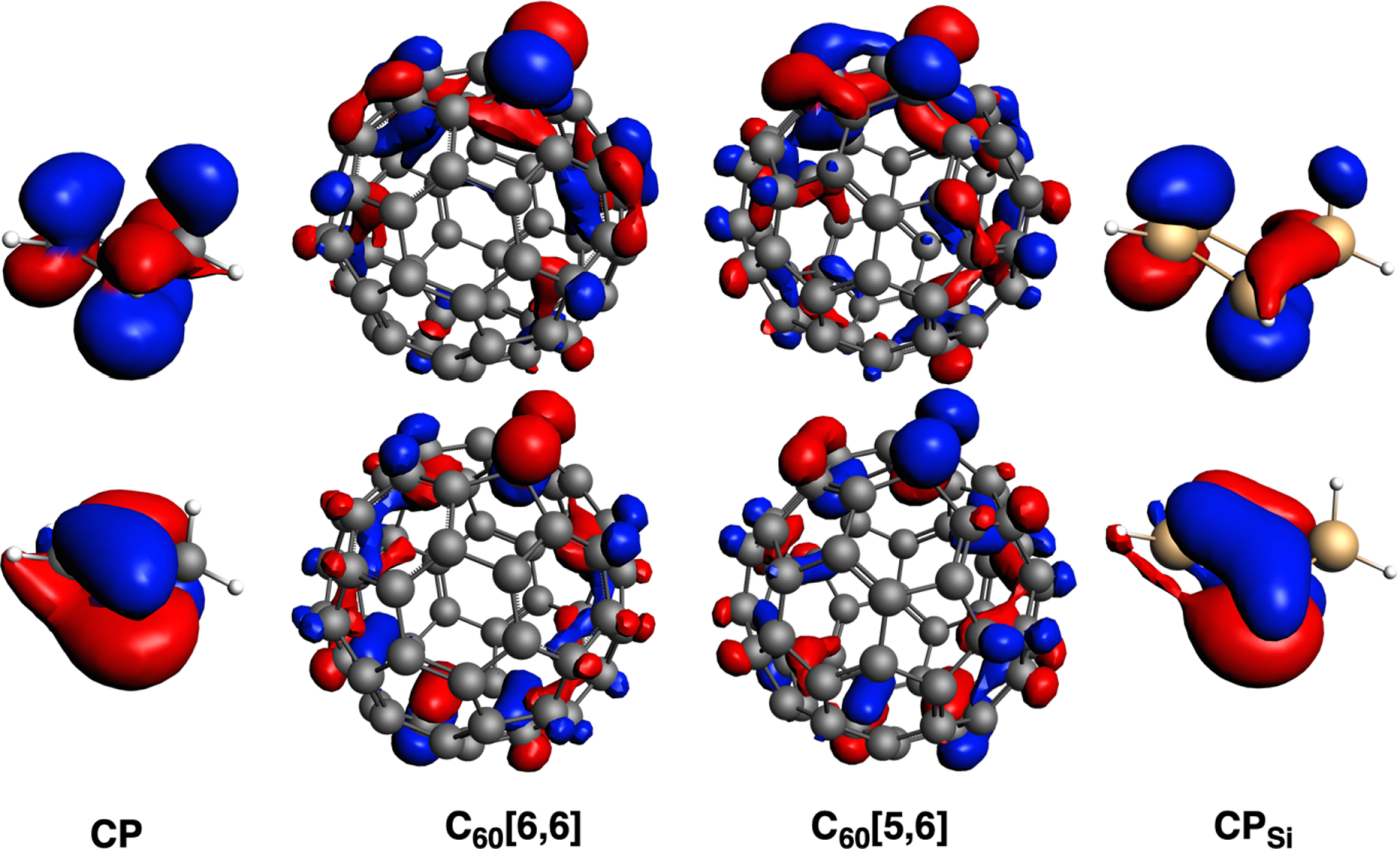
SOMOs of C_60_, CP and CP_Si_ of the fragments considered in the EDA analysis of C_60_Product [6,6] and C_60_Product [5,6], at their triplet state. Those for Li^+^@C_60_ are almost the same. These same fragments are those used for the TS, but less deformed.

At difference to C_60_, when the fullerene is exchanged by Si_60_, an intriguing observation arises: the interaction energy for the [5,6] attack is more favorable than that of the [6,6] attack by 2.7 kcal/mol, and similarly for Li^+^@Si_60_, with a difference of 2.4 kcal/mol. Noticeably, this trend becomes clearly more pronounced when considering the CP_Si_ substrate interacting with Si_60_, with an interaction energy more favorable for the product [5,6] by almost 40 kcal/mol (Table 4). The effect appears to stem not from the substrate but rather from the nature of the fullerene; as in the case of C_60_, with or without lithium, the [6,6] attack is favored by 8.1 and 8.3 kcal/mol, respectively. Such a difference in the nature of the fullerene is already supported by the different HOMO and LUMO orbitals when going from C_60_ to Si_60_ (Figure S1). Whereas in the case of the former, both HOMO and LUMO involve π molecular orbitals of the fullerene and the bonds connecting to the CP, in Si_60_, both orbitals have lobes with similar contributions for most of the Si atoms.

Examining the detailed contributions with CP_Si_ substrate, first for Si_60_, the stronger interaction of the [5,6] attack (from −128.9 to −168.4 kcal/mol) as compared to the [6,6] attack goes with a large increase of the attractive electrostatic and orbital interactions, which largely compensate the sudden increase of Pauli repulsion (from 399.1 to 630.7 kcal/mol). The main reason for the large energy differences between the [6,6] and [5,6] attacks observed for the DA of CP_Si_ to Si_60_ is due to the fact that the attack of CP_Si_ to a [5,6] bond of Si_60_ is not a DA cycloaddition but a [4+4] cycloaddition. Hence, we are comparing different reactions that behave quite differently. Finally, when the lithium cation is introduced in the Si_60_, the trends are also kept (from −119.2 to −182.4 kcal/mol), and even more pronounced. Noticeably, the two different types of reactions in the case of the CP_Si_ substrate are responsible for such changes from [6,6] to [5,6] compared to the CP substrate above (Figure 5). In particular, whereas the products with C_60_ are similar to those with CP discussed above, in the case of Si_60_, this is not the case. When the latter interacts with the CP_Si_ substrate, more Si–Si bonds are formed, driving Si_60_Product [5,6] to a stronger interaction.

**Figure 5:**
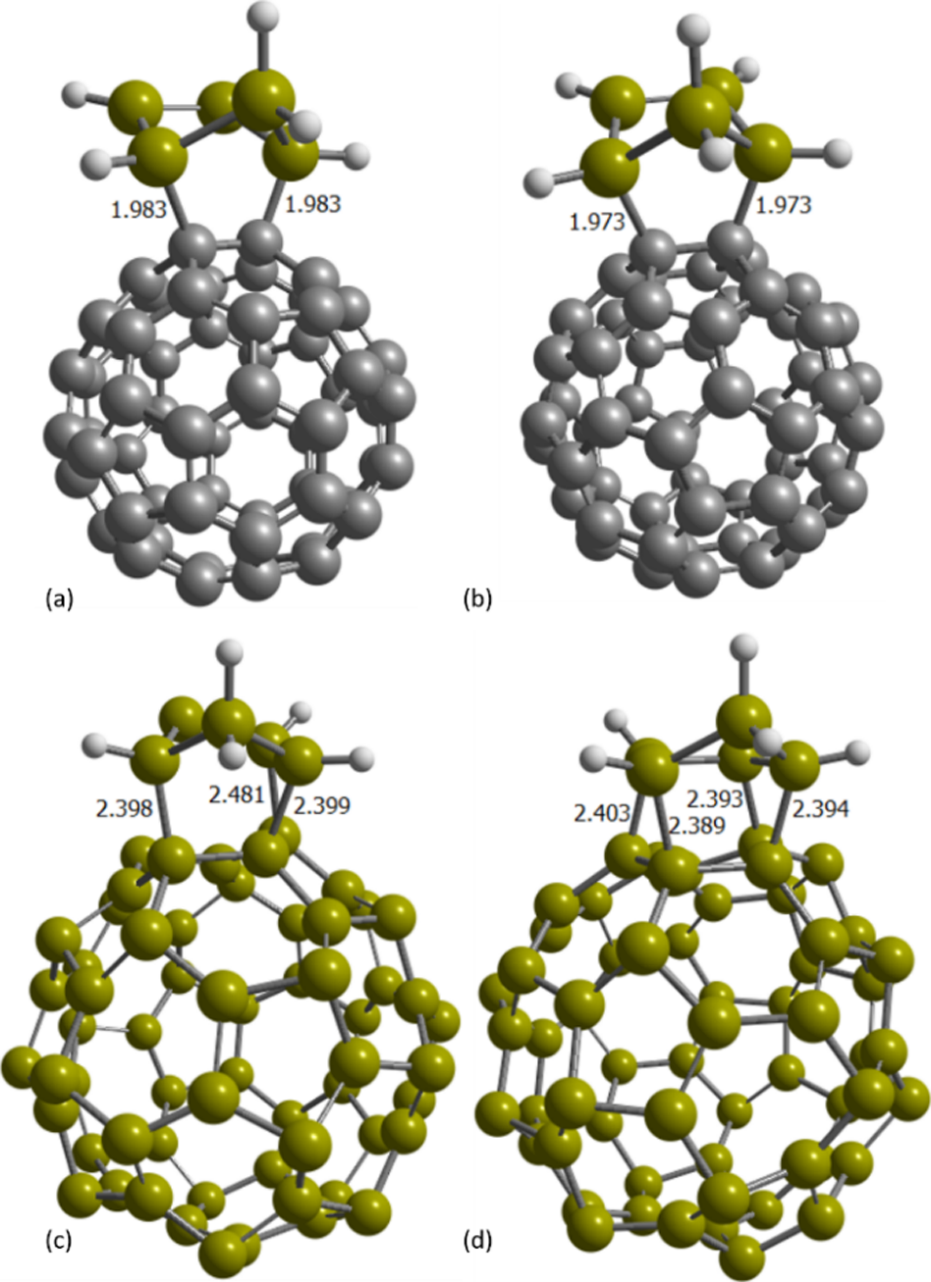
(a) [6,6] with C_60_, (b) [5,6] with C_60_, (c) [6,6] with Si_60_, and (d) [5,6] with Si_60_ products of the reaction with CP_Si_ substrate (selected distances in Å).

## Conclusions

This work elucidates the influence of structural and electronic modifications on the cycloaddition of CP and CP_Si_ to C_60_ and Si_60_. Key findings include that lithium encapsulation reduces activation barriers and enhances the thermodynamic stability of cycloaddition products, emphasizing the role of the cation in increasing fullerene electrophilicity. The [6,6] pathway remains preferred, driven by lower Pauli repulsion and stronger orbital interactions. Next, silicon substitution drastically enhances reactivity with the increase of electrophilicity. As a result, there is significant exergonic stabilization of the [5,6] pathway when reacting with CP_Si_. Lithium further boosts electrophilicity, albeit to a lesser extent than with C_60_. In addition, CP_Si_ introduces novel interaction patterns, favoring the [5,6] pathway due to the overstabilization of silylene groups at the fullerene surface.

Variations in the Pauli repulsion and electrostatic and orbital interactions explain reactivity trends across C_60_ and Si_60_ fullerenes and transition states. The asynchronic transition state and enhanced interactions account for the thermodynamic and kinetic favorability of the [5,6] pathway with silicon-based systems.

## Supplementary Material





## Notes

Absolute energies of all computed species (PDF)
XYZ coordinates (XYZ)
