# Supplementary material for: Diels–Alder Cycloaddition of Cyclopentadiene to C60 and Si60 and Their Endohedral Li+ Counterparts
Source: J Phys Chem A. 2025 Jan 27;129(5):1386–95. doi: 10.1021/acs.jpca.4c08287 (PMC12129254; doi:10.1021/acs.jpca.4c08287)
Supplement: Supplementary file 1 [file jp4c08287_si_001.pdf]

Diels-Alder cycloaddition of cyclopentadiene to C_{60} and Si_{60} and their endohedral Li^+ counterparts

Omkar Charapale,[‡] Jordi Poater,[§,*] Sergio Posada-Pérez,[‡,*] Miquel Solà,[‡,*] and Albert Poater[‡,*]

[‡]Institut de Química Computacional i Catàlisi and Departament de Química, Universitat de Girona, c/ Maria Aurèlia Capmany 69, 17003 Girona, Catalonia, Spain

[§]Departament de Química Inorgànica i Orgànica & IQTCUB, Universitat de Barcelona, 08028 Barcelona, Spain; ICREA, 08010 Barcelona, Spain

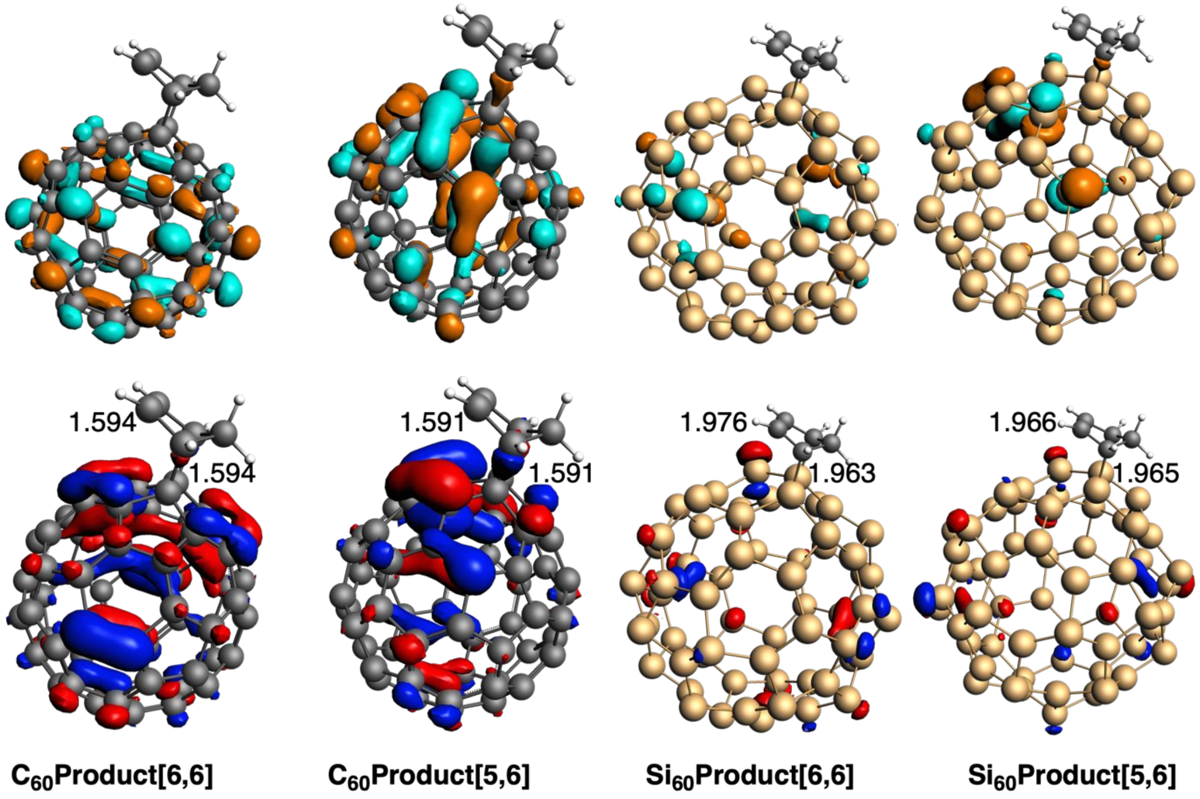

Figure S1. HOMO (bottom) and LUMO (top) of C_{60}Product[6,6] and C_{60}Product[5,6], and Si_{60}Product[6,6] and Si_{60}Product[5,6]. Bond lengths (in Å) connecting both fullerene and CP are also enclosed.
